# Supplementary figures and images for: Getting higher on rugged landscapes: Inversion mutations open access to fitter adaptive peaks in NK fitness landscapes
Source: PLoS Comput Biol. 2022 Oct 31;18(10):e1010647. doi: 10.1371/journal.pcbi.1010647 (PMC9648849; doi:10.1371/journal.pcbi.1010647)

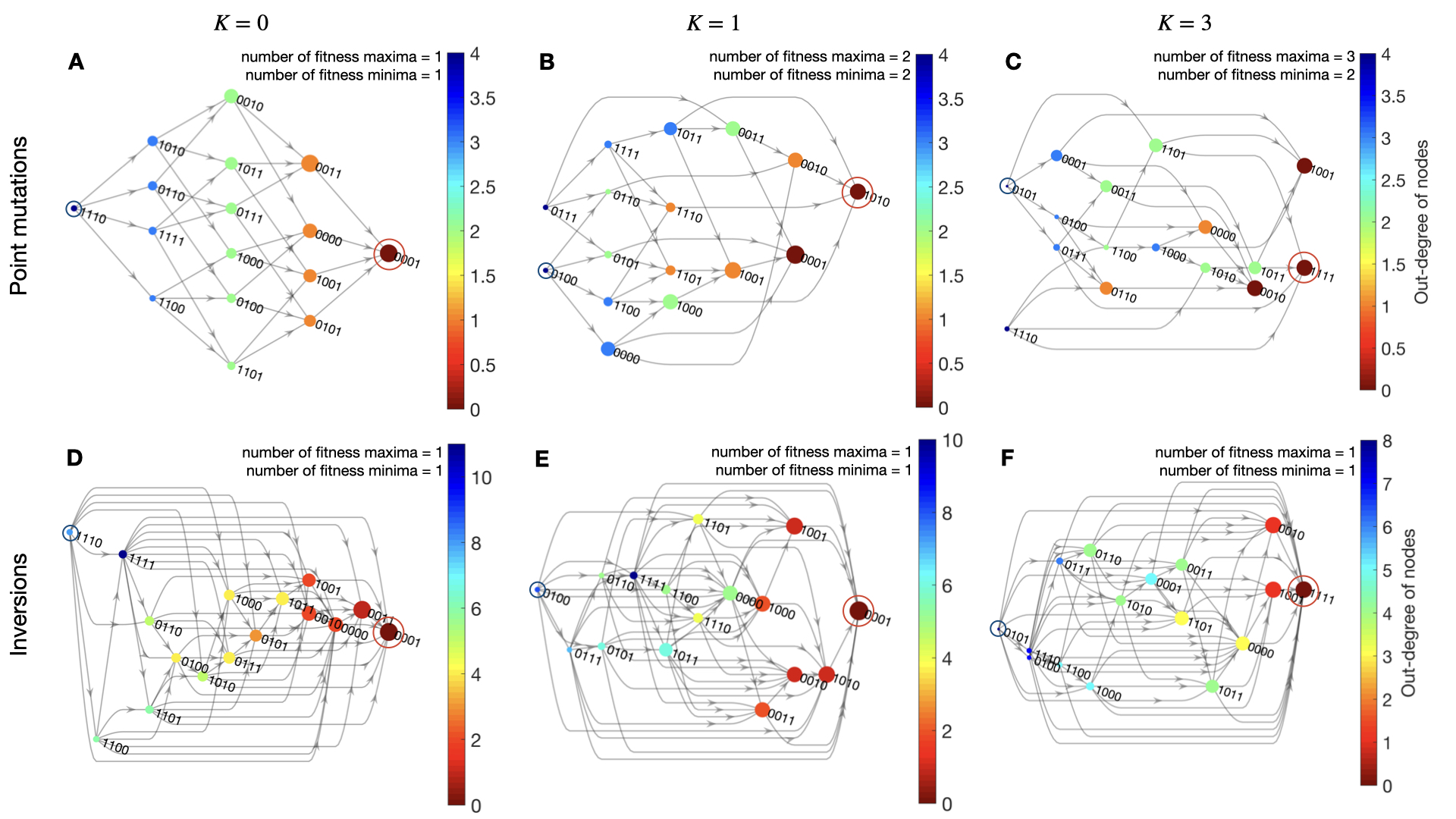

Supplement: S1 Fig — Representative instances of the NK model for N = 4 and their fitness networks in layered representation. The layers are constructed such that each node is assigned to the first possible layer, with the constraint that all its predecessors must be in earlier layers. The colors of the nodes correspond to the values of the out-degrees, i.e. the number of edges going out of a node (note that color scales differ in range between panels). Therefore, nodes with node out-degrees equal to zero correspond to local fitness maxima (sink nodes). The landscapes’ ruggedness are: single peaks K = 0, intermediate ruggedness K = 1 and full rugged case K = 3, for adjacent neighbouring epistatic interactions. Node sizes are scaled with fitness values (best fitness, largest and vice versa). Global maximum of fitness are encircled in red. While the global minimum in blue. The total number of fitness maxima and minima are also reported. See 3 in main text for epistatic interactions with random neighbouring. (TIFF) [file pcbi.1010647.s001.tiff]
