# Supplementary material for: Getting higher on rugged landscapes: Inversion mutations open access to fitter adaptive peaks in NK fitness landscapes
Source: PLoS Comput Biol. 2022 Oct 31;18(10):e1010647. doi: 10.1371/journal.pcbi.1010647 (PMC9648849; doi:10.1371/journal.pcbi.1010647)
Supplement: S1 Text — (PDF) [file pcbi.1010647.s002.pdf]

S1 Supporting Text

Contents

1 Fraction of invariant inversions **2**

2 Linear or limited sized inversions **3**

2.1 Bounded sized inversion 3

2.2 Linear chromosomes 6

3 On the synergistic effect of inversions in the adjacent neighbourhood **7**

1 Fraction of invariant inversions

To enumerate the average number of invariant inversions, let us characterise what an invariant inversion is.

An inversion of size j on a sequence $x_i...x_{i+j-1}$ is invariant if and only if:

$$x_i...x_{i+j-1} = \bar{x}_{i+j-1}...\bar{x}_i \tag{1}$$

Thus, $\forall m \in [0, j], x_{i+m} = \bar{x}_{i+j-1-m}$ and if j is an odd number, then for $m = \frac{j-1}{2}$ we have $x_{i+m} = \bar{x}_{i+m}$. This is not possible, hence only even sized inversion can be invariant.

Then, given a random inversion of even size $j = 2 \times q$, what is the probability that this inversion is invariant?

For any sequence, $x_i...x_{i+q-1}$ there is only one sequence $x_{i+q}...x_{i+j}$ such that the property (1) is respected. Thus, the probability for an inversion of even size $j = 2 \times q$ to be invariant in a binary alphabet is $\frac{1}{2^q}$.

Let us note the event: "a random inversion is invariant on a binary sequence" as R^2 and "a random inversion of size j is invariant on a binary sequence" as R^2_j. By cumulating all the possible inversion sizes for a genome of size N we get :

$$\begin{aligned}
\mathcal{P}(R^2) &= \sum_{j=1}^{N} \mathcal{P}(\text{size} = j)\ \mathcal{P}(R^2_j |\ \text{size} = j) \\
&= \sum_{j=1}^{N} \frac{1}{N} \mathcal{P}(R^2_j |\ \text{size} = j) \\
&= \frac{1}{N} \left[\sum_{q=1}^{\lfloor \frac{N}{2} \rfloor} \frac{1}{2^q} + \sum_{q=0}^{\lfloor \frac{N+1}{2} \rfloor} 0 \right] \\
&= \frac{1}{2N} \frac{1 - 1/2^{\lfloor \frac{N}{2} \rfloor}}{1 - 1/2} \\
&= \frac{1}{N} \left(1 - \frac{1}{2^{\lfloor \frac{N}{2} \rfloor}}\right) \\
&\underset{n \to +\infty}{=} \frac{1}{N}
\end{aligned}$$

It is worth noticing that small inversions are the main drivers of invariability.

Similarly, it can be proven that for a four-bases alphabet, $\mathcal{P}(R^4) \underset{n \to +\infty}{=} \frac{1}{3N}$.

2 Linear or limited sized inversions

To complement our analysis, here we present the results of adaptive walks simulations for inversion mutations with different constraints: bounded maximum size and linear chromosome. The simulation setup is the same as that used in the calculations of the mean fitness shown in Fig 4 of the main manuscript.

2.1 Bounded sized inversion

We simulated inversion mutations with an upper limits $s \leq N$ that ranges from 1% of chromosome size —a single locus (i.e. inversions are like point mutations)—up to 100% of chromosome size. Figures Fig A and Fig B shows eight examples of maximum inversion mutations sizes $s = \{1, 2, 4, 8, 16, 32, 64, 100\}$, for a genome of size $N = 100$ and for adjacent and random epistatic neighbourhood respectively. The mean fitness is averaged over 100 instances of adaptive walks simulations, for all values of epistatic interaction, $K = \{0, 1, \dots, N-1 = 99\}$.

In agreement with Fig 4, for $K = 0$ (no epistatic interaction between neighboring loci) the average fitness for all inversions sizes are equal, i.e. $\langle f \rangle_{K=0} \simeq 0.667$. This is also consistent with the analytical result, $\langle f \rangle_{K=0} = \frac{2}{3}$, for smooth landscapes with a single peak [1, p. 55]. For $K > 0$, the average fitness $\langle f \rangle_K$ increases with K until reaching a maximum value of fitness, and then decreases towards a minimum at $K = N - 1$, with a really similar behaviour than in Fig 4. Given that when the maximum inversion size is $s = 1$, the only possible mutation is a point mutation and when $s = N$ all the inversions are available, a smooth transition from the blue line to the red one is expected, and indeed observed. Also, consistently with what we observed on Fig 4 for the random epistatic neighbourhood, in this case no difference of fitness is observed for low K values, whatever s (Fig B).

However, importantly, the gain of fitness due to the increased s value is not linear, for example: for low values of K, in Fig A, the transition from $s = 1$ to $s = 2$ correspond to an average final fitness gain comparable to the transition from $s = 4$ to $s = 100$. This non-linearity on low values of K fades away when K reaches very high values (typically higher than $K = 80$). Indeed, for such high values of K the correlation between the fitness of a genome and its mutants is very low (See roughness in Fig 5). In this case, the final fitness reached becomes mainly a combinatorial challenge, and the combinatorics of available mutations increases with s.

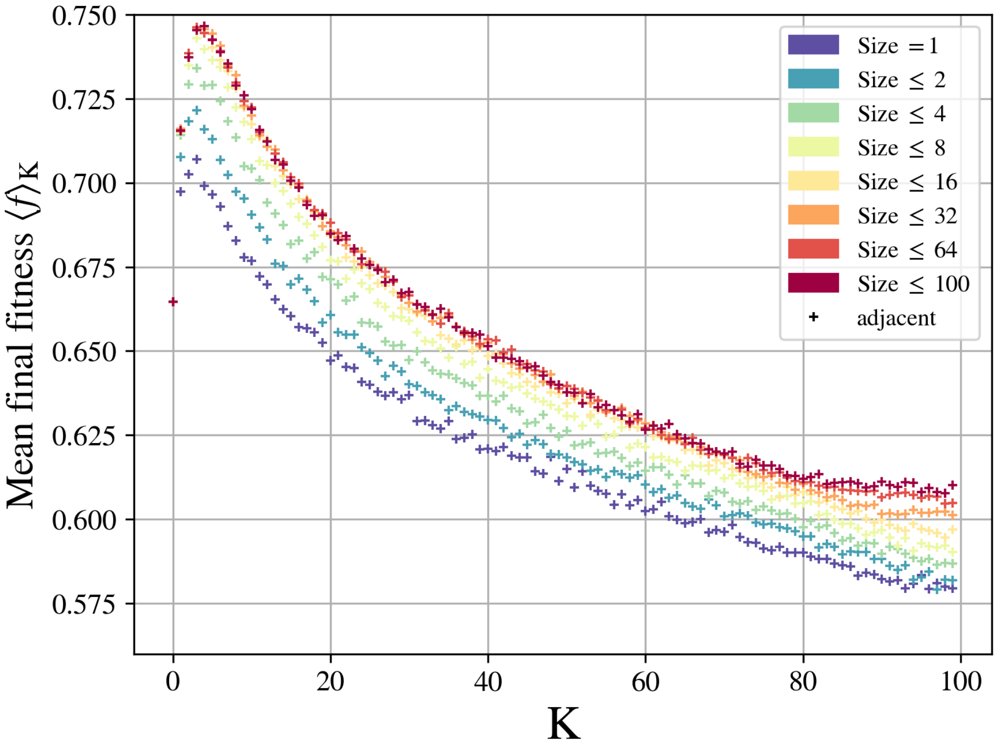

Fig A: **Mean final fitness for inversion mutations restricted to a certain size range (epistatic interaction with adjacent neighborhood).** Changes in mean final fitness for different epistatic parameter K for point mutations (blue) and inversions restricted to a certain size range (sizes values and colors nomenclature are displayed in the figures), averaged for 100 instances of adaptive walks simulations in NK landscapes.

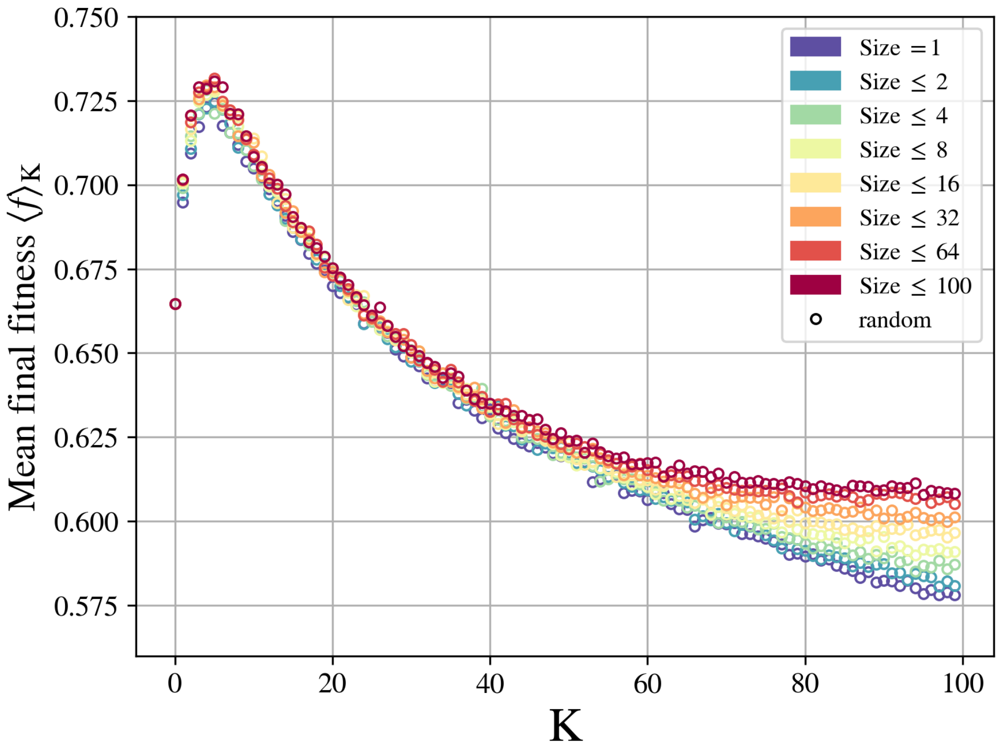

Fig B: **Mean final fitness for inversion mutations restricted to a certain size range (epistatic interaction with random neighborhood).** Changes in mean final fitness for different epistatic parameter K for point mutations (blue) and inversions restricted to a certain size range (sizes values and colors nomenclature are displayed in the figures), averaged for 100 instances of adaptive walks simulations in NK landscapes.

2.2 Linear chromosomes

Linear chromosomes imply a limitation of the general inversion on circular genome. All inversions are allowed, except the ones overlapping with the boundaries. A direct consequence is that inversions of larger size are more likely to cross the boundaries and thus are more likely to be forbidden. The figure Fig C shows the average final fitness when considering only linear inversions for all K values and for the two epistatic neighbourhood (green marks). To ease the comparison with the general case, the final fitness with inversions on a circular genome and with point mutations is presented in shaded red and blue respectively The values are relatively similar to inversions, the main difference being around $K = N - 1$. This behaviour is the same as the one observed previously with bounded inversions (with an upper size limit between $s = 32$ and $s = 64$). This is consistent with the idea that the presence of inversions in linear chromosomes mainly affect large sized inversions.

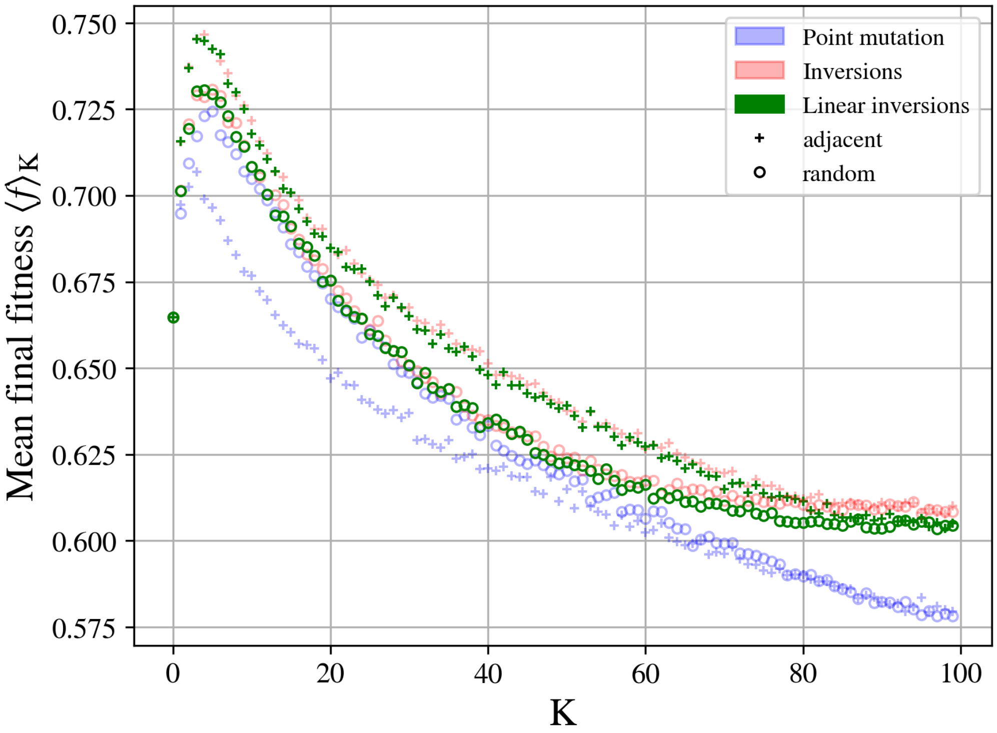

Fig C: **Comparison of mean final fitness between circular and linear chromosomes.** Changes in mean final fitness for different epistatic parameter K for linear chromosomes (green markers), averaged for 100 instances of adaptive walks simulations in NK landscapes. For comparison with circular genomes, the initial values are shown dimly for inversions (shaded red) and point mutations (shaded blue). The circle (respectively cross) markers correspond to random (respectively adjacent) neighbouring epistatic interactions.

3 On the synergistic effect of inversions in the adjacent neighbourhood

Simulation of inversion mutations on the NK fitness landscape show that this operator enables reaching higher peaks than point mutations, especially for large values of K (typically $K > 40$, for $N = 100$). However, for lower values of K, this result only holds for the adjacent epistatic neighbourhood but not for the random epistatic neighbourhood (in that case, the fitness reached by inversions being similar to the fitness reached by point mutations). This is likely to be due to the fact that inversions effect is confined to a segment (we call inversions "segmental operators"). When epistatic interactions are also confined to a segment close to the focal nucleotide (which is the case for the adjacent neighborhood but not the random one), both segments can largely overlap, hence limiting the effect of the inversion to a set of epistatically interacting genes. Although a full mathematical proof is out of the scope of this paper, we develop here a representative mathematical analysis that illustrates this point.

To develop our argument, first let's recall Eq. 8 (main text). In an NK fitness landscape, the fitness $f(\mathbf{x})$ is given by:

$$f(\mathbf{x}) := \frac{1}{N}\sum_{i=1}^{N} f_i(x_i; x_{i_1}, \ldots, x_{i_K}). \tag{8}$$

Let us consider a genome $\mathbf{x} \in \{0,1\}^N$ with size $N = 12$ as an illustrative example that we can handle:

$$\mathbf{x} = x_1x_2x_3x_4x_5x_6x_7x_8x_9x_{10}x_{11}x_{12}, \ \forall x_i \in \{0,1\}.$$

For simplicity, we also assume that the genome is circular (i.e. with periodic boundary conditions such that for example $x_{12+1} = x_1$, $x_{12+2} = x_2$ or $x_{1-1} = x_{12}$, $x_{1-2} = x_{11}$ and so on). Now suppose that an inversion is (randomly) located between loci x_3 and x_5, i.e. $s = 3$. Therefore $x_3x_4x_5 \to y_3y_4y_5$ and the mutated genome is (red color highlights the mutated loci):

$$\mathbf{y} = x_1x_2y_3y_4y_5x_6x_7x_8x_9x_{10}x_{11}x_{12}, \ \forall x_i, y_i \in \{0,1\}.$$

For K = 0 (no epistatic interaction), it is trivial that only three fitness contributions per loci would change.

$$\begin{aligned} f_3(x_3) &\to f_3(y_3),\\ f_4(x_4) &\to f_4(y_4),\\ f_5(x_5) &\to f_5(y_5). \end{aligned}$$

Therefore, the fitness variation of the mutated genome $\mathbf{y}$ depends on the size of the mutation s.

Here are three examples of the parameter K for an adjacent neighborhood (let us remember that for $K \neq 0$, the fitness contribution at the ith locus depends upon itself and on K other loci, and that, for the adjacent neighborhood, these K loci are located just downstream of the focal locus):

1. For $K = 1$, only the following four local fitness contributions change:

$$\begin{aligned} f_2(x_2, x_3) &\to f_2(x_2, y_3),\\ f_3(x_3, x_4) &\to f_3(y_3, y_4),\\ f_4(x_4, x_5) &\to f_4(y_4, y_5),\\ f_5(x_5, x_6) &\to f_5(y_5, x_6), \end{aligned}$$

   Note that not only do loci within the inversion contribute, but also, as a consequence of the epistatic interaction pattern, two other loci, x_1 and x_2, outside the mutated segment of the genome also contribute to the fitness of the mutant genome $f(\mathbf{y})$ (x_1 because of epistatic effect of x_3 on this locus, x_2 because of its own epistatic effect on x_3 and x_4).

1. For $K = 2$, five local fitness contributions change

$$\begin{aligned} f_1(x_1, x_2, x_3) &\to f_1(x_1, x_2, y_3),\\ f_2(x_2, x_3, x_4) &\to f_2(x_2, y_3, y_4),\\ f_3(x_3, x_4, x_5) &\to f_3(y_3, y_4, y_5),\\ f_4(x_4, x_5, x_6) &\to f_4(y_4, y_5, x_6),\\ f_5(x_5, x_6, x_7) &\to f_5(y_5, x_6, x_7). \end{aligned}$$

2. For $K = 3$, we can verify that

$$\begin{aligned} f_{12}(x_{12}, x_1, x_2, x_3) &\rightarrow f_{12}(x_{12}, x_1, x_2, y_3), \\ f_1(x_1, x_2, x_3, x_4) &\rightarrow f_1(x_1, x_2, y_3, y_4), \\ f_2(x_2, x_3, x_4, x_5) &\rightarrow f_2(x_2, y_3, y_4, y_5), \\ f_3(x_3, x_4, x_5, x_6) &\rightarrow f_3(y_3, y_4, y_5, x_6), \\ f_4(x_4, x_5, x_6, x_7) &\rightarrow f_4(y_4, y_5, x_6, x_7), \\ f_5(x_5, x_6, x_7, x_8) &\rightarrow f_5(y_5, x_6, x_7, x_8). \end{aligned}$$

2. For $K = 4$, we can verify that

$$\begin{aligned} f_{11}(x_{11}, x_{12}, x_1, x_2, x_3) &\rightarrow f_{11}(x_{11}, x_{12}, x_1, x_2, y_3), \\ f_{12}(x_{12}, x_1, x_2, x_3, x_4) &\rightarrow f_{12}(x_{12}, x_1, x_2, y_3, y_4), \\ f_1(x_1, x_2, x_3, x_4, x_5) &\rightarrow f_1(x_1, x_2, y_3, y_4, y_5), \\ f_2(x_2, x_3, x_4, x_5, x_6) &\rightarrow f_2(x_2, y_3, y_4, y_5, x_6), \\ f_3(x_3, x_4, x_5, x_6, x_7) &\rightarrow f_3(y_3, y_4, y_5, x_6, x_7), \\ f_4(x_4, x_5, x_6, x_7, x_8) &\rightarrow f_4(y_4, y_5, x_6, x_7, x_8), \\ f_5(x_5, x_6, x_7, x_8, x_9) &\rightarrow f_5(y_5, x_6, x_7, x_8, x_9). \end{aligned}$$

   In this case the four loci x_{11}, x_{12}, x_1 and x_2 are outside the mutated segment, and the total number of fitness contribution per locus that were modified by the mutation is 7.

From the above examples we can infer that, with an adjacent epistatic neighbourhood, the number of loci contributing to the variation of fitness after an inversion of size s is equal to $K + s$. On the other hand, if the epistatic links are randomly assigned according to a uniformly distributed probability, the pattern of epistatic interaction becomes more complex. For example, the mutated base y_3 can change the fitness value of any f_i (the fitness contribution of locus i). Hence, for small values of K the number of fitness contributions that will be changed by an inversion is of the order of $K \times s$ which is much higher than for adjacent epistasis $(K + s)$.

Given that, when close to a peak, in average the more fitness values are changed, the less correlated the fitness of the genome after the mutation will be compared to the genome before the mutation, the worst the mutation (because the values of f_i are drawn in a random uniform distribution between 0 and 1 and the total fitness is greater than 0.5). This explains why, for low K values, the roughness (local standard deviation of the fitness values) of the landscape if different for the two different epistatic neighbourhood (see Fig 5). Now, of course, the maximum number of locus contributing the fitness change after an inversion is N. Hence, for high K values, this number saturates and the difference between the two neighbourhood vanishes.

References

1 Kauffman SA. The origins of order: Self-organization and selection in evolution. Oxford University Press, USA; 1993.
