# Supplementary material for: Getting higher on rugged landscapes: Inversion mutations open access to fitter adaptive peaks in NK fitness landscapes
Source: PLoS Comput Biol. 2022 Oct 31;18(10):e1010647. doi: 10.1371/journal.pcbi.1010647 (PMC9648849; doi:10.1371/journal.pcbi.1010647)
Supplement: Attachment — Submitted filename: Responses_to_the_reviewers_and_list_of_changes.pdf [file pcbi.1010647.s003.pdf]

Responses to the reviewers

We appreciate the careful and exhaustive reading of the manuscript and the points addressed by the reviewers, which have been very useful to us. Below, we quote the reviewers' comments followed by our point-by-point responses (in blue). To fully respond to comments, we performed several computer simulations and, as a complement to our manuscript, we now include a supporting text (S1 Text) and figure (S1 Fig). The changes to the main manuscript are indicated in blue text in the new version.

Reviewer 1:

> "In this manuscript, the authors investigated how populations can explore the genotypic space using the fitness landscape framework. The usual approach to this question is to use point mutations. However, the authors chose here to use a different type of mutations: small structural variants and inversions in particular. Including these larger mutations change the connectivity of the genotype space, allowing for "long range" jumps across the genotype space and therefore the fitness landscape. In particular, it means that such variants are more effective at exploring the fitness landscape and less likely to end up on a local fitness pic. The authors therefore present here an alternative explanation to the classical problem of "crossing fitness valleys"."
> I enjoyed reading this manuscript. The idea explored here is really interesting.

We thank you for the detailed review of our manuscript and for the insightful comments and questions. It is very encouraging for us that you enjoyed reading our manuscript and that our idea was interesting for you. We address your points below.

Major comments:

> **1.1** "One question I have that is not answered is how the choice of modelling a small circular chromosome (at most 100 bp) affects the results obtained here. Using a linear chromosome should reduce the connectivity between genotypes, but it is unclear how strong the effect would be."

Yes, as the reviewer rightly deduces, the connectivity between genotypes is reduced for linear chromosomes. In the linear case the number of accessible mutants is smaller than the case circular sequences. This is because the enumeration of inversion mutations is smaller than in the circular case. We have carried out simulations of adaptive walks with linear chromosomes on the NK landscape and we verify that the results on the expected fitness values are similar to those of the case of circular chromosomes. Thus, the analysis and conclusions of our work are correctly confirmed. Therefore, the reported results do not depend on our choice to work with circular chromosomes. This point addressed by the referee is discussed in Section 2 (T3 Fig.) in the supporting text S1 Text. In response to this comment we have edited the main text to remark this point (lines 126-127 and 161-163).

> **1.2** "I could not find any information on the distribution of inversion length used for the simulations. To my understanding the simulations were conducted allowing the inversions to span from 1 bp to N bp. I wonder what happens if the inversion size were restricted to a certain size range (e.g. 10 to 30% of the chromosome)."

We thank the reviewer for this comment because we believe that our argument for inversion mutations as a mechanism to escape local fitness peaks is now much more complete. To answer the point made by the reviewer, we ran several computer simulations for different representative sizes of inversion lengths, and a detailed discussion on the effects of inversion sizes is now presented in Section 2 of the supporting text S1 Text. The results show that the larger the length of the inversion mutations, the higher the expected fitness values (see figures T1 Fig and T2 Fig in S1 Text). See lines 280-289 in the main text.

> **1.3** "The authors are using the weak mutation strong selection approximation. This means that a mutation that has an infinitely small chance of appearing, will still appear with certainty if it is the only beneficial mutation left. This assumption means that the exploration of the fitness landscape under this regime and a more "realistic" exploration of the fitness landscape may yield different results. In particular, since the connectivity of the fitness landscape increases (a lot) in the presence of inversions, it means that the ratio beneficial/deleterious mutations is much smaller for genotypes close to the optimum (than in the point mutation scenario – as it has access to many more "bad" options). I therefore wonder how the use of inversion may affect the waiting time for beneficial inversions, compared to point mutations. Therefore I believe it will be interesting to characterize the distribution of fitness effects of both SNPs and inversions, and the waiting time to the next "step"."

We thank the reviewer for this important point. However, we shall stress that the distribution of the fitness effect is not constant over evolutionary time. By definition, at the moment of convergence, all mutations are deleterious or neutral. As suggested by the reviewer to distinguish the effect of inversion and point mutation, we characterize the DFE in random sequences against K, and for the random and adjacent neighborhoods of epistatic interaction. Unfortunately, as we can see in figure R1, the results obtained do not show a clear difference between the different types of mutation. We regret not being able to give a conclusive answer to this point pointed out by the reviewer.

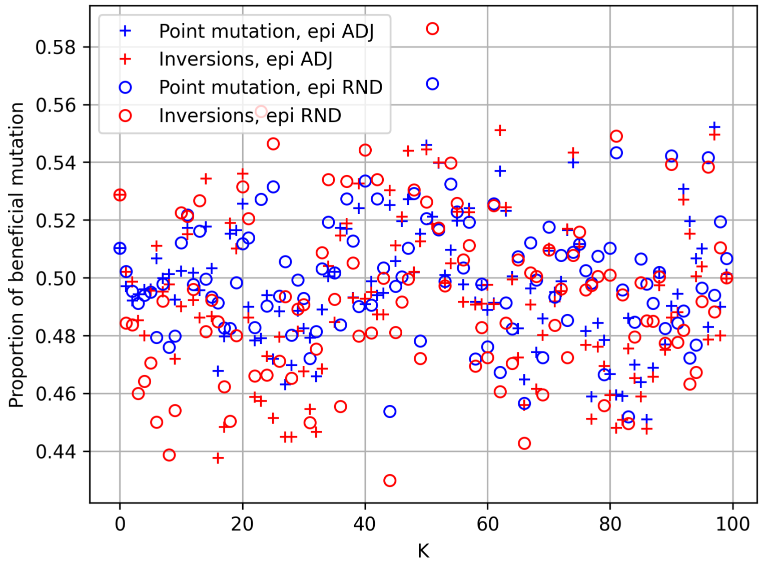

R1: **Relation between beneficial and deleterious mutations.** Proportion of beneficial mutations vs the epistatic parameter K for point mutations (blue) and inversion mutations (red) averaged for 100 instances of adaptive walks simulations.

1.4 "It would also be interesting to compare the dynamics of the two populations (one using point mutations, the other inversions), and see which one increases first in fitness, using parameters similar to WMSS regime – but allowing populations to naturally evolved. For competition, it is better to be fitter now than to be the fittest latter. Therefore, while the process described here is interesting, it is hard to judge its evolutionary relevance without this information."

We greatly appreciate this comment from the reviewer and agree that it would be interesting to compare the consequences of point mutations and inversions on a population scale. However, this point involves analyzing the time between mutational events and the sweep to fixation in the population. In the scenario proposed by the reviewer, we must consider that point mutations and inversions are not population-exclusive. To correctly answer this point made by the reviewer it would be necessary a complete analysis of how the populations evolve in time where the mutation events are no longer separated. This is a very interesting perspective that is part of our ongoing research. The topic has been very interesting for us, for example, preliminary results show us that, as a consequence of the joint action between inversion mutations and point mutations, the evolutionary dynamics has two temporary scales of short periods of fitness increases alternated with long periods of minimal variability in fitness [arXiv.2105.05520]. See lines: 446 and 457 in the main text.

1.5 "Finally, one biological aspect that is ignored here is that the new mutations, generated by the inversion, are not random and are conditioned by the ancestral state. For example, the three stop codons will stop being stop codons if inverted, and a leucine, phenylalanine and a serine will become stop codons to point out the most obvious example. I believe exploring this potential aspect is beyond the scope of the manuscript but it would be interesting if the authors could comment on this, as the NK model may fail to capture this effect."

We thank the reviewer for this important remark. We have followed the reviewer's suggestion and highlighted this point in the manuscript (lines 461-474).

1.6 "I believe Figure 3 would be better served by showing less cases (moving them to a supplementary figure) but with remaining figures better annotated. In addition, according to the legend "Node colours correspond to fitness values, increasing from left to right.", so both colors and positions along the x axis indicate fitness? In that case, I do not understand why points of different colors are on the same vertical line."

This is indeed a very good suggestion. Now we show fewer cases, we also include the number of local peaks and minima in order to guide the reader to the most significant difference between the graphs for point mutations and the graphs for inversions. We apologize for the misunderstanding about the colors nomenclature and the legend. This motivated us to rethink a better way to convey the main message. Now the colors of the nodes correspond to the value of the out-degree of a node. We believe that this allows direct identification of fitness maxima in the network. Also to avoid confusion, we have explicitly commented on how the layers are build in the graphs: each node is assigned to the first possible layer, with the constraint that all its predecessors must be in earlier layers. To appreciate the variations in fitness per node, we now show the sizes of the nodes scaled with their fitness value (i.e. greater the fitness value greater the diameter of the node and vice versa). The other cases are now shown in supporting figure S1 Fig. See lines: 289-213 in the main text.

Minor comments:

> **1.1.1** "Line 92: this is a bit misleading here. The only example given above of a submicroscopic inversion was the 7bp inversion in the mitochondria. Reference 54 here refers to inversions that are between 1 and 3kb. It is roughly 3 orders of magnitude of difference."

Thanks to the referee for indicating this point. We have corrected the references according to the sentence related to intragenic mutations. We have also edited and reorganized the introduction section with the purpose of presenting more clearly the length scales involved in inversion mutations. Hopefully this illustrate better the biological framework of our work. See lines 54-55 in the main text.

> **1.1.2** "Line 134-135. Should N not be excluded in the inequality? If the inversion spans the whole chromosome, then 5'-3' becomes 3'—5' and vice versa and nothing has changed."

This is a good point made by the referee, since the expression, $0 \leq d_H(\mathbf{x}, \mathbf{x}') \leq N$, takes into account the viability of all mutations (mathematical combinations). But just as it was written, it is true that, when $d_H(\mathbf{x}, \mathbf{x}') = N$, omits that it changes everything and nothing from a biological point of view. In response to reviewer's point we have added a comment on this in the manuscript (see lines 78-80).

> **1.1.3** "Line 200-201. I do not understand this sentence."

The reviewer is right and we appreciate very much pointing out our mistake. We have changed the sentence and we hope that now the message is clearer. See lines 145-147 in the main text.

Reviewer 2:

> "Trujillo et al. present an interesting generalization of the study of adaptive walks in which the main mechanism of producing variation is through inversion mutations instead of point mutations as usual.
>
> As we know the adaptive walk framework holds in the strong selection weak mutation regime. In this limit, adaptation is modelled as the exploration of the genotypic space through point mutations, and once a local optimum of the fitness landscape is reached the process is halt. Conceptually, the problem of the adaptive walk modifies as the mechanism of variation changes. The definition of local optimum is made with respect to the mechanism of genetic variation.
>
> Through extensive computer simulations, the authors show that higher fitness values are reached through inversion mutations. The effect is even more prominent when epistasis occurs among adjacent locus of the genome.
>
> I think the authors bring an important contribution to this topic. Certainly, this contribution will shed new light into this problem and motivate future contributions of the concerning other mechanisms of genetic variation. I think the contribution can be accepted for publication after some important points are raised in a revised version of the manuscript."

We thank you for the review of our manuscript and for the insightful points. We also appreciate your positive feedback. For us it is a great stimulus in our purpose of modeling and understanding the consequences of chromosomal rearrangements and the perspectives that this implies. We address your points below.

> " Points to be addressed:
> In order to understand the effectiveness of the mechanism of inverse mutations when compared to point mutations, we need a fair comparison of the problems that are conceptually distinct, as highlighted above. Important measurements to be considered and compared:"
>
> **2.1** "What is the density of local optima under the mechanism of inversion mutations for the same set of parameters used for point mutations (same N, same K)? It is important to the get these measurements for the two cases."

We thank the referee for this important point. The referee's question is correct, but (computationally and mathematically) hard to answer. One strategy is, for example, to identify in the fitness network those nodes whose out-degree was equal to zero, which corresponds to a fitness maximum, for both inversions and point mutations. Which, for a genome space $\{0,1\}^N$, one has to compute the degree of 2^N nodes, and at the same time there are at least N point mutations or N^2 inversions per node (for $N = 100$ the number of nodes is $\sim 1.3 \times 10^{30}$). Therefore an exhaustive numerical analysis to rigorously answer this question is beyond our resources. For point mutations, the problem of calculating the number of local maxima has been extensively studied mathematically (Weinberger, Phys. Rev. A (1991)). For example, the two well known cases are: *i*) the trivial case with $K = 0$, which is a landscape with a single peak, and *ii*) when $K = N - 1$, where the expected number of local maxima is $2^N/(N+1)$. On the other hand, the mean number of maxima grows asymptotically as $\sim (2\lambda_K)^N$ with a constant λ_K that increases with K. (See for example, Hwang, Schmiegelt, Ferretti and Krug J. Stat. Phys. (2018); [arXiv:1708.06556]. Therefore, after studying the literature related to this problem, we conclude that a mathematical analysis for inversion mutations is beyond the central purpose of our current manuscript. Therefore, unfortunately, we are not in a position to be able to respond to the referee in a satisfactory manner. However, as an illustration, in figure R2 we present an example for a genomes with sizes $N = 7$ and 10. In these figures we can verify that the mean number of local maxima for inversion mutations is smaller than the mean number of maxima of point mutations. We presume that this difference will persist for any value of N.

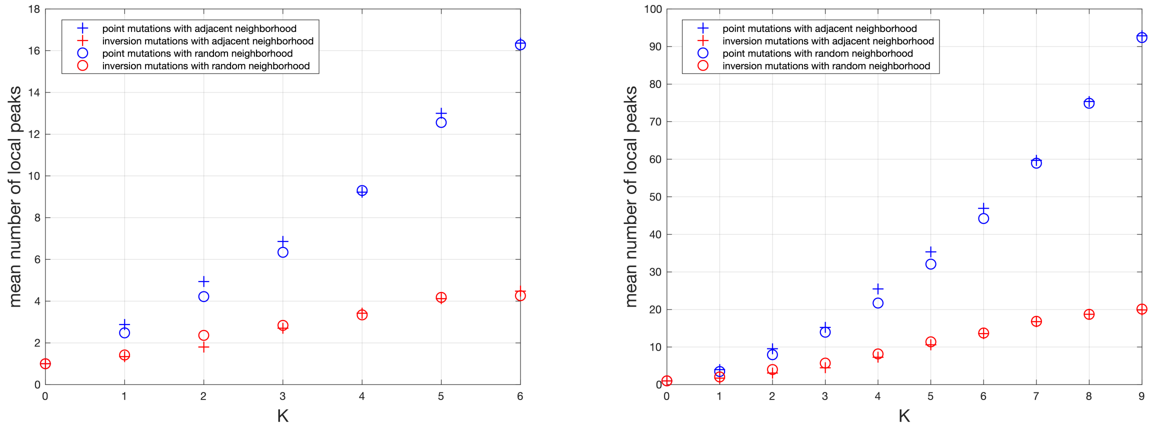

R2: **Mean number of local peaks.** Analysis of the number of local peaks for two small genotypes of sizes $N = 7$ (left panel) and $N = 10$ (right panel), vs thee epistatic parameter K.

2.2 "My impression is the that the resulting fitness landscape under inversion mutations is much smoother than that found under point mutations. Please try to compare the outcome of the adaptation process in a scenario in which the ruggedness of the fitness landscapes under the two scenarios are similar. Does this explain the pattern found in Figure 4?"

Assuming that smoothness means fewer peaks in the fitness landscape and according to the previous point, the referee's intuition seems correct: The number of peaks seems to be lower for inversion mutations, so the fitness landscape should be less rugged. However, the question about the comparison of adaptive processes for two scenarios with similar landscape ruggedness is not very clear to us. If by similar we assume to mean a similar number of average peaks between the case of point mutations and inversion mutations, that would imply different values of K, hence different fitness landscapes, implying that adaptive processes are inherently different. On the other hand, the explanation of the pattern of the average fitness curves as a function of K can be explained in the complexity catastrophe scenario exposed by Kauffman in The origins of order: Self-organization and selection in evolution, and also as a consequence of the epistatic interaction neighborhood pattern. That is, the computation of fitness for each genome depends on how the calculation of fitness contribution per locus is defined in the NK model (Eq. 8 in the main manuscript). The increase in epistatic interactions affects the adaptive trajectories on the fitness landscape, in such a way that an increase in the number of epistatic interactions K implies a greater restriction of the fitness values that can be reached through selection. This difficulty becomes manifest in a decrease of the expected fitness values. However, despite the catastrophe of complexity, inversion mutations can explore mutations that eventually allow to escape from a local fitness peak and thus achieve average fitness values that are generally higher than those of point mutations. In response to the reviewer's point we have included a more detailed mathematical

analysis of an illustrative instance to explain the pattern found in Figure 4 (see Section 3 in supporting text S1 Text.)

> **2.3** "What is the main reason underneath a no noticeable variation of the average fitness with the mechanism when random neighbouring epistatic interactions are assumed (up to K = 40)?"

We are deeply grateful to the referee for this point because it allowed us to be aware this particular aspect of the NK model that are often assumed to be understood, but are not necessarily so. We argue that this unnoticed variation between the fitness values of point mutations and inversions for $K \simeq 40$ is due to the fact that inversions are *segmental* operators. That is, they operate on a specific length of the genotype, covering a domain of epistatic interactions that will depend on whether the neighborhood is adjacent or random. In response to the reviewer's point we discuss this issue in the manuscript, and a illustrative mathematical argument is presented in Section 3 of the supporting text S1 Text. (See lines 348-364 in the main text).

> **2.4** "Is that possible to generalize the definition of roughness of the landscape along a given evolutionary trajectory, as found in "Aita T, Iwakura M and Husimi Y 2001 A cross-section of the fitness landscape of dihydrofolate reductase Protein Eng. 14 633"?"

We are very grateful to the referee for pointing out this question. Throughout the first version of our manuscript, we had focused on the *topological* aspects of fitness and mutational networks, but given the difficulty in characterizing the density of the fitness maxima (as illustrated above), we haven't a *topographical*–like measure to differentiate how inversions change the fitness landscape relative to point mutations. We follow the reviewer's suggestion and study the definition of roughness proposed by Aita, Iwakura and Husimi in Protein Engineering, Design and Selection (2001). As suggested by the reviewer, in the new version of the manuscript we introduce a generalization of the roughness measure. We expanded the results section presenting the analysis of the local roughness difference between point mutations and inversions. (See lines 319-364 in the main text).

Reviewer 3:

> "In this paper the authors study how structural variants, more specifically very small inversions, affect the topology of the fitness landscape and how this in turns affect the adaptive walks. This is a very relevant piece of information as most theory focus on single step mutations, and ignores the impact of larger types of mutations. The results obtained suggest that by changing the connectivity between genotypes, inversions allow to cross fitness valleys and reach higher fitness peaks, reducing the number of times a walk ends up in a local fitness maxima.
>
> I think this work is highly relevant and important and is an important step to introduce other type of mutations in fitness landscape theory. I really liked the idea behind the paper, but it was a bit complicated to follow sometimes, and so most of my comments focus on how the manuscript is written and I also have a few questions about the methods. I also have some minor comments that are at the end of the review."

We thank you for the very detailed review of our manuscript and for the insightful comments and suggestions. We also appreciate your positive comments. We edited the manuscript and did our best to make it more readable. We address your points below.

Major comments:

> **3.1** "The introduction could be shortened and go straighter to the point. Right now, it reads more as a historical introduction, and at the same time speaks very little of the work done (theoretical and empirical) on the impact of different types of mutations on the fitness landscape and the genotype to phenotype map (e.g. Aguilar-Rodríguez et al Evolution 2018, Alejandro V. Cano and Payne 2020 Plos Comp Biol, Zheng et al Science 2019 and see a review of some papers in Kemble et al 2019 Evolutionary applications). In addition, it would be good to explain why inversions are of interest. Maybe give some biological examples to contextualize the reader."

We thank the reviewer for these suggestions. We edited the introduction and shortened it and now it's organized to be more straight to the point. We thank the reviewer for these excellent references and we have included them. As the genotype-phenotype map is not a topic on which the work focuses, we have eliminated the phrase that referred to it in the initial version of the manuscript. Therefore, reference Kemble et al. (2019) was not cited. Also following the reviewer's suggestion, the introduction was rewritten to better present the biological framework of our work.

3.2 "The whole manuscript needs a revision to make it more focused, including the result section which is quite difficult to navigate. The theme at hand and the results obtained are not easy to explain, but although the subsections of the results make sense, there seems to be a lot of side thoughts throughout the results, which complicate readability."

We are very grateful to the reviewer for the suggestions made so that we can hopefully achieve a more readable manuscript. We revised the whole manuscript and we did our best to make it more articulated.

3.3 "The first point of the results does not correspond to new results per se, so it should be in the methods section. I understand the need for the preamble, but it belongs in the methods section."

The reviewer is right on this point. So, we moved this part to the methods section.

3.4 "I could not find the size of the inversions studied. This information should be easily accessible in the method description. In addition, it would be important to know the impact of the inversion size on the dynamics of the landscape."

We thank the reviewer for this remark. This point is concomitant with the second point addressed by the first reviewer. Now the information about the sizes of the inversion mutations is included in the manuscript.

3.5 "I do not understand the use of the conjugation to generate the inversion. Permutation should be enough to emulate the inversion since it corresponds to a change in the nucleotide order."

We respectfully wish to make the point that an inversion mutation is not simply a permutation between bases or "a change in the nucleotide order" as the reviewer says. An inversion *is a segment of the sequence* that is flipped (inverted arrangement) relative to the normal orientation (standard arrangement). Therefore, our decomposition in a conjugation (or flip) operation per locus and then a permutation between loci is equivalent to the flipped segment of an inversion mutation. In response to the referee's point we added the following remark in the introduction section (lines 42–43): "... models generally focus on their effect on synteny but neglect the possibility that inversions disrupt coding sequences [37]".

3.6 "The title does not reflect the part of the inversions, which is really the cool thing about this work. Maybe something like "Getting higher in rugged fitness landscapes with inversions" (not very inspired I know, but it is just an example)."

We thanks the reviewer for this very important point, that was also addressed by the editor. Following their suggestion we propose the following title:

> "Getting higher on rugged landscapes: Inversion mutations open access to fitter adaptive peaks in NK fitness landscapes"

3.7 "The discussion is the most focused part of the manuscript, but I think it would be important to add two points:

- What is the impact for the accessibility and your conclusion of the sequence studied being circular vs linear?

- Is there any biological data that can support this type of result?"

We thank the reviewer for these two point. The first one is also related to the first point of the first reviewer. This point is now addressed and expanded in the Section 2 in supporting text S1 Text, where we show several simulations to answer the point. (see lines 126-127 and 161-163 in the main text).

In relation to the second point, as far as we have been able to know, unfortunately we do not have data with which we can directly confront our model. In fact, the phenomenology of intragenic inversions does not appear to be much documented or even empirically analyzed. But nevertheless, there is evidence of intragenic inversions that supports the phenomenology proposed in our model (this is commented on in the manuscript in the introduction and discussion sections).

3.8 "Finally, please revise your writing style as it overall seems too colloquial (I confess that I like it, because it shows the enthusiasm behind some of the results, which I share, but it is not the usually accepted one in journal articles)."

We thank the reviewer for drawing our attention to the style of the manuscript. We greatly appreciate that the reviewer likes our style and shares our enthusiasm. So, we have worked on the text together with the help of two native English speakers, whom we thank for their contributions to the manuscript. We have done our best to address each of the points raised by the reviewer.

Minor comments:

3.1.1 "Please revise/tone down the use of the term "Let us ..." "

We appreciate this point. We have now taken care about the use of the term "Let us ...".

3.1.2 "L2: Persuasive is a rather uncommon term for the fitness landscape metaphor, may be a more used one could visual or appellative."

We changed the word to "influential" which corresponds to what we want to convey and is also often used in literature.

3.1.3 "L9: I like the informal style, but maybe use only note, instead of let's notice."

We made the change suggested by the reviewer.

3.1.4 "L103: To sharpen the context → It is not clear what is meant here. Is it to contextualize?"

We made the change: "To sharpen the context..." → "It is worthwhile..."

3.1.5 "Line 144: I find it weird the use of a scheme with numbering like an equation instead of a figure with both schemes, but I am not sure what are the policies of the journal regarding this."

It is a mathematical notation that allows us to formally illustrate the mutation operations that we study in this work. Above all, we did it for the purpose of making it very explicit that a reversal mutation is not a mere permutation of sites on a chromosome.

3.1.6 "L162: Table (capitalize the t)."

We thank the reviewer for this important detail. We made the change.

3.1.7 "L200: "Both graphs are not equivalent, although classical reasoning based on point mutations overshadows their differences!" –¿ Please clarify this sentence."

We thank the reviewer for pointing out our mistake. We have changed the sentence and we hope that now the message is clearer. See lines 145-147 in the main text.

3.1.8 "L258: 259 - Since this is not always the case, maybe it would be better to add either: "Generally," or "In most of the cases". This way the reader knows that exceptions happen as is seen in figure 3."

We thank the reviewer for this suggestion. We made the change for "In most of the cases..."

3.1.9 "L487 – schema –¿ schematic no?"

We thank the reviewer for pointing out this point. In fact, we change it to "representation", which we consider to be more appropriate.

3.1.10 "In the figures (to make the life easier to the reader) please put the name of the parameters (and then the symbols in parenthesis)."

Excellent point from the reviewer. We apologize for our "minimalist" notation in the figures. We follow the referee's suggestion and take care that the names are explicit.

3.1.11 "Table 1: please explain the meaning of each column name."

We made the reviewer's suggestion.

Description of changes made to the manuscript

We made several changes in the manuscript, in particular:

1. We changed the title of the manuscript to "Getting higher on rugged landscapes: Inversion mutations open access to fitter adaptive walks in NK fitness landscapes".
2. We modified the introduction of the manuscript, which is now shorter. We direct the introduction more clearly towards the biological context of inversion mutations.
3. The results section changed quite a bit:
   (a) The "Limits in the single-nucleotide mutation scenario" subsection has now been placed in the methods section.
   (b) As a consequence of the above, the introductory paragraph located between lines 64 and 69 changed.
   (c) Now a remark is included on what happens when the inversion's size is equal to the size of the genotype. Lines 78–80.
   (d) The legend of Table 1 was edited to better inform about the variables reported.
   (e) Between lines 106 and 127 the text was edited to clarify some aspects of the consequences in the enumeration of mutations for the cases of linear and circular sequences.
   (f) Between lines 145 and 149 the sentences were rewritten to make their meaning clearer.
   (g) Fig. 3 is now splitted to make it more accessible to the reader, showing fewer cases and better detailing what the figures show. We now include a supporting figure (S1 Fig) as a complement of the new version of Fig. 3. Therefore, and to convey the message more clearly, the text of the manuscript changed between lines 189–213.
   (h) The remarks made by the reviewers led us to realise that inversions are not commutative and in lines 214 and 219 we comment on the effects of this aspect.
   (i) Between lines 280–289 we now include information on the analysis of linear and circular chromosomes, of inversions with upper size limit. Also, this new information is supplemented in more detail in the new supporting text S1 Text.
   (j) An important group of new results are included in the new version of the manuscript (lines 319–364), which are summarized in the new figure 4. Roughness measurements are reported, showing the differences between point mutations and inversions. The mathematical definition of the roughness measure that we have used is presented at the end of the methods section.
4. We revised the discussion according to the new positioning of the manuscript. In particular, we addressed the two points raised by reviewer 1 (comparison of the evolutionary dynamics with inversions vs. point mutations; generalization to more bio-like sequences).
5. The following references are now included in the new version:

18. Aguilar-Rodriguez J, Peel L, Stella M, Wagner A, Payne JL. The architecture of an empirical genotype-phenotype map. Evolution. 2018;72(6):1242–1260.
19. Zheng J, Payne JL, Wagner A. Cryptic genetic variation accelerates evolution by opening access to diverse adaptive peaks. Science. 2019;365(6451):347–353.
20. Cano AV, Payne JL. Mutation bias interacts with composition bias to influence adaptive evolution. PLoS computational biology. 2020;16(9):e1008296.
52. Aita T, Iwakura M, Husimi Y. A cross-section of the fitness landscape of dihydrofolate reductase. Protein engineering. 2001;14(9):633–638.
80. Rutten J, Hogeweg P, Beslon G. Adapting the engine to the fuel: mutator populations can reduce the mutational load by reorganizing their genome structure. BMC Evolutionary Biology. 2019;19(1):1–17.
81. Beslon G, Liard V, Parsons DP, Rouzaud-Cornabas J. Of evolution, systems and complexity. In: Evolutionary Systems Biology. Springer; 2021. p. 1–18.
85. Barabási AL, Stanley HE. Fractal concepts in surface growth. Cambridge university press; 1995.
