# Supplementary material for: Getting higher on rugged landscapes: Inversion mutations open access to fitter adaptive peaks in NK fitness landscapes
Source: PLoS Comput Biol. 2022 Oct 31;18(10):e1010647. doi: 10.1371/journal.pcbi.1010647 (PMC9648849; doi:10.1371/journal.pcbi.1010647)
Supplement: Attachment — Submitted filename: Responses_to_referees_2.pdf [file pcbi.1010647.s004.pdf]

Second round of responses to reviewers

We appreciate the careful and exhaustive reading of the manuscript and the points addressed by the reviewers, which have been very useful to us. Below, we quote the reviewers' comments followed by our point-by-point responses (in blue). The changes to the main manuscript are indicated in blue text in the new version.

Reviewer 1:

> The authors have addressed the majority of the comments from the reviewers. The manuscript is now much clearer.
> After reading this manuscript, I realize there is one major underlying issue that was not raised in the first time. All of the theoretical and simulations work was done assuming a binary alphabet for the nucleotides. While this is a an extremely common and accepted approach, I believe it comes with an extra set of caveats here in this specific context (intragenic inversion, where the complementary sequence matters). Indeed, for the normal points mutations system, all genotypes that are separated by a hamming distance of 2, can be reached via 2 mutations, regardless of the size of the alphabet used. This no longer holds true for the inversion mutation system presented here for example AAAT and AAGG also have a Hamming distance of 2, yet they cannot be accessed with any amounts of inversion mutational steps (and inversion size) – something that a binary alphabet cannot capture. Based on this idea, under a 4 letter-alphabet, the better connectivity that fitness landscapes display through these intragenic mutations will be clearly reduced, with even some section being completely inaccessible from others. How this will affect the results described in the manuscript is however impossible to predict.

We appreciate that you find our manuscript to be more readable now. We are also deeply grateful for your detailed review of our manuscript and the important issue you addressed regarding the binary alphabet used in our model and how the case of a 4-letter alphabet might affect our results. In the present version of the manuscript we address this point in the discussion section (see lines: 488-516):

> All throughout this study, we focused on binary sequences, simplifying the 4-bases nature of real genomes. Although the binary description is very common in theoretical and computational models, it is important to mention that the properties of the different mutational operators, hence of the generated mutational networks, may differ depending on the size of the alphabet [73]. In the case of inversions, although our main conclusions about the complex structure of the mutational network still hold, a 4-bases alphabet with two pairs of complementary bases (`A-T` and `C-G`) would introduce important properties compared to the binary case. Indeed, given the mathematical definition of inversions (Eq. 5), it is straightforward that the composition of the inverted segment will conserve the relative fraction of `AT` and `CG` pairs relatively to the original one (a specific situation being the inversion of a segment of size one that can only switch `A` and `T` or `C` and `G`). It immediately follows that inversions cannot change the `AT`/`CG` ratio of the sequence and that, for a sequence of length N, the mutational network generated by inversions contains at least $N+1$ disconnected sub-networks (which sizes will depend on the `AT`/`GC` ratio of the sequence, strongly biased sequences leading to smaller sub-networks). Hence, compared to the binary model, the 4-bases model increases the size and connectivity of the mutational network generated by point mutations [73]. On the opposite, in the inversion-generated mutational network, it isolates several sub-networks from each others. The effect on evolutionary dynamics, as we studied it here using the NK landscape, is still to be explored. Indeed, depending on the composition of the initial sequence, with a 4-bases alphabet some local/global optima may not be accessible. Consequently, the advantage of inversion mutations may be reduced, and even be cancelled if the number of local optimum is very low (i.e. for $K \ll N$). However, it is worth mentioning that real genomes undergo both kinds of mutational events and that point mutations connect the inversion-isolated sub-networks by changing the `AT`/`GC` ratio of the sequence. Exploring how both kinds of mutations (and others) interact is clearly beyond the scope of this manuscript, but studying the synergistic effect of inversions and point mutations in a more sequence-realistic model like the Aevol model [80,81] is clearly an appealing perspective.

Minor comments:

> **1.1** "At the beginning of the discussion (and conclusion), I believe it would be useful to remind the reader that the authors are talking about intragenic inversion. Because the phenomenon

described here does not apply to larger inversions (to my understanding), which is what most people thing of, when mentioning inversions."

We thank the reviewer for this point. We have made the change and specified that we are talking about intragenic mutations (see lines 385, 535 and 537).

1.2 "L 6-8: "Within this framework, the evolution of any population can be conceptualised as adaptive walks driven by successive mutations constrained by incremental fitness selection." Evolution can also arise from random walks on a flat fitness plateau."

The reviewer is right on this point. We have now emphasized that adaptive walks can be driven by successive mutations constrained by incremental or neutral fitness steps (see line 22).

1.3 "L42: I would be more precise here. Most models have focus on large inversions, where the main type of sequence disruption is caused by the break points themselves, which to rend the affected gene(s) non-functional. The kind of change the authors refer to here is unique to the extremely small inversions investigated here (and that's why they are not included in the more general inversion models)."

We thank the reviewer for this important point. To address this point, we have changed the sentence to emphasize that, as models generally focus on very large inversions (typically larger than a single gene), therefore they include their effect on synteny (or deleterious effects on breakpoints), but neglect the possibility that small inversions may occur inside the coding sequences. (lines: 57-58).

1.4 "L55: "Recombination is not considered, so we are modelling asexual replication." The causality in this sentence has been reversed. I would suggest editing it to "We are modelling asexual replication, therefore recombination is not considered.""

We thank the reviewer for pointing out our error in the causal order between asexual replication and recombination. We changed the sentence (see lines: 71-72).

1.5 "L304-305: I am not sure the use of the word "verify" here is correct. If the authors simply want to describe the figure, maybe the use of "observe" or "display" would be clearer. Alternatively, if the authors are checking some theoretical prediction against the simulations results, then a reference to said prediction would be really helpful."

We made the change suggested by the reviewer (see lines: 320-321). The observation made by the reviewer is correct. In fact, at this point in the paragraph we are describing the behavior of the simulation results. The verifiable fact with the theory had already been done at the end of that paragraph citing tables 2.1 and 2.2 of Kauffman (1993).

Reviewer 2:

"The authors had satisfactorily addressed the points raised in my report. New material was added, and particularly, measures of roughness of the fitness landscape upon inversions are now presented. In my opinion the manuscript is suitable for publication in its present form".

We thank you very much for the review of our manuscript and for the insightful points. We deeply appreciate your point about roughness measures of the fitness landscape, which allowed us to show an important aspect of inversion versus point mutations.

Reviewer 3:

"I really like this manuscript and I think the authors made a very good job in answering all reviewers comments and incorporating them in the manuscript. I have only small comments here and there. The only problem was that in the clean version I was not able to see the figures and there was no legend for figure S1, but I am not sure if this was due to the formatting issue or not. All lines in the comments refer to the tracked version (where I could see the figures)."

We thank you for the very detailed review of our manuscript and for the insightful comments and suggestions. It is a pleasure for us that you liked our manuscript and for your positive comments about our work. We address your comments below.

Minor comments:

3.1. "L190 - The reference to the figure with epistatic interactions (which I guess is figure S1) is missing."

Yes, it refers to figure S1 Fig. We have solved this by including this supporting figure at the end of the main script to avoid this kind of problem when compiling the TeX file (see line 206 and caption of Fig. 3).

3.2 "L228 - we want to verify if (instead of that)."

We made the correction pointed out by the reviewer (see line: 244).

3.3 "L232 - Remove the Now in the beginning of the sentence."

We made the correction pointed out by the reviewer (see line 248).

3.4 "closets → closest :p"

Thanks for pointing out this dyslexic mistake (see line 264).

3.5 "L284:285 - Maybe say it the other way around: Simulations with linear chromosomes show no significant difference from our reference circular model (see supplementary S1 Text, section 2 for detailed results)."

We made the change suggested by the reviewer (see lines: 284-285).

3.6 "L317: 318 - This a weird formulation for the sentence. Maybe something like: "Therefore, our results show that in the presence of inversions it is possible to reach higher fitness when compared to adaptive walks with only point mutations."

We thank the reviewer for this remark. We made the change suggested by the reviewer (see lines: 333-334).

3.7 "L320:321 - The beginning of the sentence is weird."

We have changed the sentence (see lines: 336-339).

3.8 "L392, 430 - graph-theoretical ?"

We made the change (see the abstract, and lines 408 and 446).

3.9 "L428 - evolutive → evolutionary."

We made the change (see lines: 193, 444 and 451).

3.10 "L453: 455 - i don't understand the use of opposite in the sentence. And the following sentence is quite cryptic, what is the former and the latter? Please clarify."

We thank the reviewer for point out this problem. We changed the sentences and we hope that it is now clearer (see lines: 469-471)

3.11 "Legend of figure 3 - the reference for the figure with epistatic interactions is missing. Also it would be good to add a small sentence for people to note that the color scales differ in range between panels."

We made the correction to the reference for the supporting S1 Fig (see also the answer to point 3.1 above). We also added the sentence to note that the color scales are different between colorbars.

3.12 "Legend of figure 4 - Instead of "The behaviour of the higher reached fitness vs the epistatic parameter K" maybe something like Changes in mean final fitness for different epistatic parameters (K) for ...
For the inset legend it would be better to add that this done for random and adjacent neighbouring epistatic interactions."

We thank the reviewer for these suggestions. We changed the sentence and also included the information of neighbouring epistatic interactions.

3.13 "Supplementary text - Section 2.2 line 57 - linear inversions mainly affect → do you mean the presence of inversions in linear chromosomes mainly affects large sized inversions?"

Yes, we thank the reviewer for pointing this out. We corrected the sentence.

3.14 "Figure S1 needs a legend."

We solved this issue (see also the answer to point 3.1 above).

Description of changes made to the manuscript

1. We made several minor changes to the manuscript based on reviewers' comments. The lines where we made those changes can be identified in the responses to reviewers and have also been highlighted in blue in the main manuscript.

2. The biggest change was in the discussion section, in which we now include the point concerning four-letter alphabets in accordance with the important observation made by the first reviewer. Consequently, between lines 488 and 516 the text was edited to address this point.
